# Potential of adipose derived stem cell preparations in neurological repair and regeneration

**DOI:** 10.52601/bpr.2021.200025

**Published:** 2021-04-30

**Authors:** Laura Combes, Xenia Sawkulycz, Wen-Hui Fang, Baoqiang Guo, Mark Slevin

**Affiliations:** 1 Department of Life Sciences, Manchester Metropolitan University, Manchester, UK; 2 Department of Biological and Geographical Sciences University of Huddersfield, Huddersfield, UK

**Keywords:** Adipose derived mesenchymal stem cells, Micro-fragmented adipose tissue, Suicide gene therapy

## Abstract

Stem cell therapy is a promising treatment for neurogenerative disease as well as inflammatory and immune mediated diseases. Decades of preclinical research has demonstrated stem cell ability to differentiate into multiple cell lineages and be utilised in regeneration and repair with their immunomodulatory and immunosuppressive properties. This work has provided the fundamental scientific knowledge needed to launch various clinical trials studying stem cell therapy in autoimmune disorders, stroke, and other tissue injury. Despite the early success many of these promising therapies are yet to breakthrough into clinical use. In this review, we highlight the recent developments in the use of stem cells as therapeutic agents for neurological conditions as well as their failures and how the clinical translation can be improved.

## INTRODUCTION

A substantial social and economic burden is placed on the globe due to neurological disorders. Neurological disorders can take many different forms and can present in many different ways. There is a real need for new treatment strategies to tackle these complex diseases with currently no cure for many forms. One potential therapeutic which is being researched is cell based therapy (Srijaya *et al*. 2014). Stem cells are seen as a therapeutic in themselves, but research suggests they can be a useful drug carrier. The research into drug carriers is essential as many different drugs have the ability to treat different diseases. However, the nature of the drug and the human body over time degrades the compounds, the immune system is activated and clears the drug out the system, or the drugs become so dilute that it is ineffective. The use of stem cells as a drug carrier that could be vital to transport the drug to target regions and control the environment (Srijaya *et al*. 2014).

Stem cells are currently used to deliver cancer drugs and treat several different cancers, including lung adenocarcinoma, glioblastoma, and leukaemia. MSCs have been engineered to express enzymes with the abilities to convert non-toxic prodrugs into cytotoxic products, in tumour models they have been shown to localize to tumour tissue where this conversion takes place leading to the damage of tumour cells. This therapy also known as suicide gene therapy (SGT) that has led to preclinical and clinical trials for the treatment of glioma (Aboody *et al*. 2013). The main enzyme used in these trials was cytosine deminase (CD) which converts the prodrug 5-flurocytosine into its toxic variant 5-fluorouancil (5FU). Two different CD proteins have been researched in SGT, CD/5FC bacterial (bCD) and CD/5FC yeast (yCD). Although both catalyse 5FU in similar ways, they have different efficiencies as yCD displays thermal instability (Kievit *et al*. 1999) directly inhibits nucleic acid synthesis and DNA metabolism, these traits mean that 5FU has been used in anticancer treatments previous however the lack of direct targeting limited the use. This initial concept was proven in animal models and then in small scale clinical trials. Which demonstrated the conversion causes the inhibition of glioblastoma cell growth. The same successes could not be replicated in large scale clinical trials leading to a decrease in further development of SGT. The shortcomings of the therapy have since been adapted by further research in particular thermal instability was mended by protein engineering in the yCD gene (Korkegian *et al*. 2005).

HSV encodes a TK gene that functions differently to human thymidine kinases (hTKs) and more efficiently catalyses prodrugs. The resulting products are incorporated into DNA strands during replication and prevent strand pairing in actively proliferating cells. Most importantly in this toxicity for normal cells is prevented as there is no interference with hTKs. Multiple purine and pyrimidine analogues are compatible with HSV-TK including ganciclovir (GCV), ACV, brivudine (BVDU) and valganciclovir (valGCV).

A trial using (HSV-TK) which converts to monophosphorylate GCV to produce the cytotoxic triphosphate ganciclovir (GCV-TP) was effective (Li *et al*. 2005). The killing of the cells is mediated by proliferating cells meaning highly proliferating tumours are killed much faster than slower proliferating tumours. Again, this limited success led to disappointment mostly as a result of low transduction efficiency. Further, more recent work demonstrated the inability of retroviral vectors to efficiently transduce quiescent cells (Hossain *et al*. 2019). A new lentiviral SGT has proven successful in animal model to both transduce quiescent cells and achieve complete remission of GBM (Hossain *et al*. 2019). This new treatment with valGCV is safe for long term treatment meaning it can effectively eliminate tumour cells not sensitive to the shorter GCV treatment. Early success in animal models needs to be further explored in human clinical trials.

Although we have seen major advances in SGT therapy many of the larger phase III clinical trials have failed. It is important to note that the early success of treatments in animal models needs to be revisited for much of these xenografts don’t effectively mimic the aggressive and invasive nature of many cancers especially glioblastoma. The use of new humanised rodent systems could provide more valuable information and improve translation to trials.

## MESENCHYMAL STEM CELLS AND THEIR POTENTIAL IN NEUROLOGICAL REGENERATIVE MEDICINE

**Figure 1:**
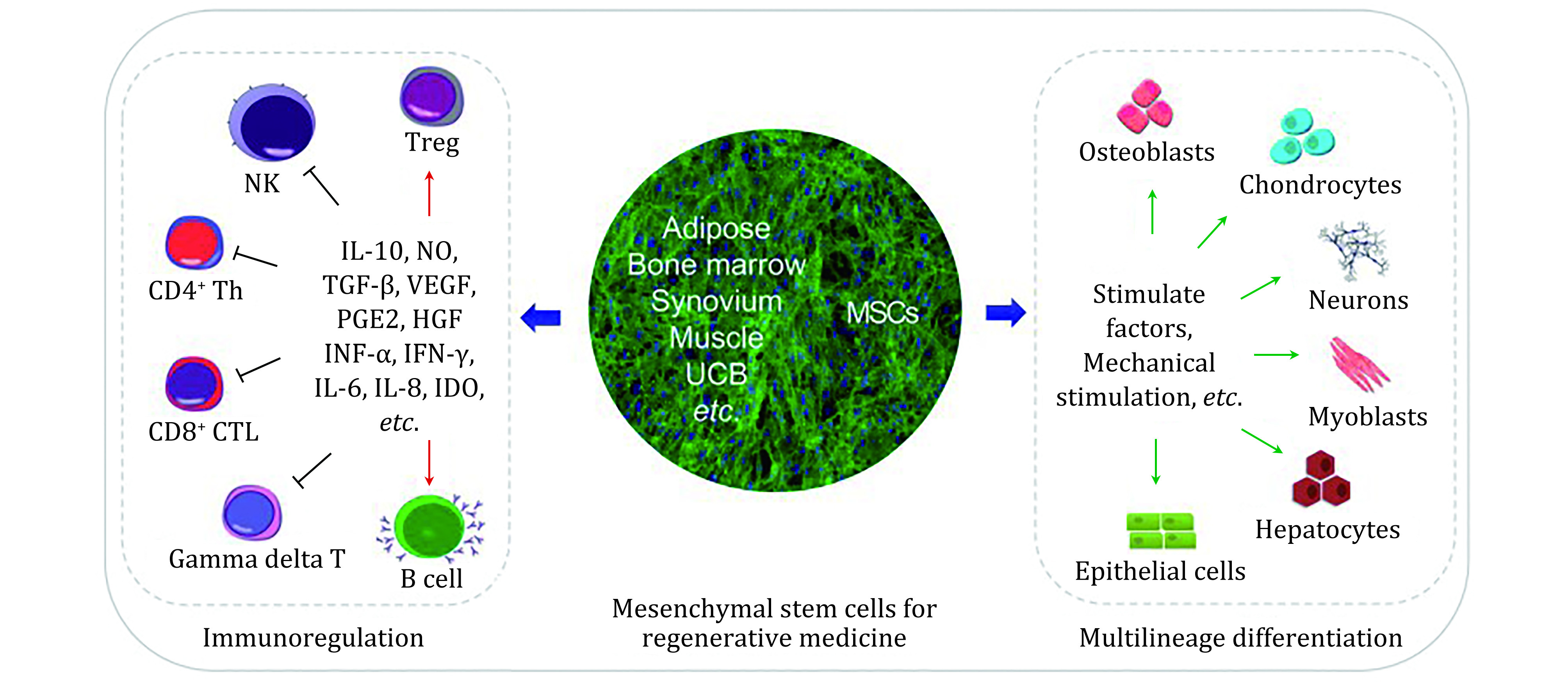
Schematic diagram showing MSCs in terms of regernative medicine. MSC extracted from multiple sites and can differentiate into multiple linages and have display immunoregulatory properties (Han *et al*. 2019)

MSCs have shown great potential for the treatment of many diseases, and over the past decade have become the most used cellular therapy in research. The use and application of MSCs has been beneficial in regeneration medicines though the significant development of molecular and transplantation techniques (Han *et al*. 2019). The potential of these cells stems from the ability of MSCs to home to specific areas of damage, potential to differentiate into several cell lineages as well as secreting factors of proliferation (Fig. 1) in the treatment of neurological diseases, there is a promising approach to cellular therapies. MSCs can be harvested from several different regions, with the most popular being bone marrow and adipose tissue (Fitzsimmons *et al*. 2018). MSCs have a regenerative potential by repairing neural tissues (Momin *et al*. 2010), it has been demonstrated that the conditioned media of MSCs facilitates the recruitment of endothelial cells and speeds up the healing process.

A particular interest has arisen in human-adipose derived mesenchymal stem cells (hADMSCs). hADMSCs are found in abundance in adipose tissue. These particular cells are available in large volumes and can be harvested using non-invasive techniques. hADMSCs also can self-renew and differentiate into different cell types, including neurocytes, chondrocytes, and osteocytes (Miana and Prieto González 2018). When administered in the presence of brain injury, they have been shown to release neurotrophic factors. Neurotrophic factors are vital for the development of mature neurons to help them differentiate and survive (Hsuan *et al*. 2016). These cells are an essential tool in tissue repair and regenerative medicine. Despite the growing evidence to support the considerable potential of MSC-therapy, there has been little movement from “the bench to the bedside.” The initial promise of trials has often led to disappointment with inconsistent results and very few therapies making it to market (Coccè *et al*. 2019). Despite being the largest experimental cell platform, there is still no FDA approval for the use of these cells in the United States or Europe. Globally they have limited approval for the treatment of graft versus host disease (GVHD) in children in Japan, Canada, and New Zealand. With this limited exception, MSCs are still only available through clinical trial mechanisms. A query of the international database Clinicaltrials.gov found that to date, there had been over 800 trials conducted using MSCs for therapy in a variety of conditions from osteoarthritis, cancer, and stroke. More detailed queries show that of these 837 trials, only 19 were phase III trials, of which only ten had been completed meaning the further nine were either withdrawn or ongoing. The *in vitro* work on MSC therapy has provided great promise in multiple diseases, yet the limited number of phase III trials highlights the somewhat failure of this therapy to be translated into human subjects quite as successfully.

## THE USE OF MSC AS A POTENTIAL THERAPEUTIC IN NEUROLOGICAL CONDITIONS

Previous clinical trials have shown that stem cell therapy is feasible, safe, and can improve recovery. However, it must be noted that these trials will have varied characteristics of patients, dosage, and cell type delivery but overall have been deemed suitable (Bang 2016). Although the use of MSCs hasn’t quite reached expectations, there are examples of great success in several phase III trials. The first ground-breaking trial was that of Prochymal for GVHD treatment, this trial was initially taken place in 2009 (NCT00366145). In the trial a total of 240 patients were enrolled ranging from six months to 70 years. The MSCs were sourced from healthy volunteers up to 10,000 doses per donor, and were then thawed with Prochymal at the point of administration whereby two million cells per kg were administered twice a week for four weeks. Response rates were measured against placebo at 82% and 73% respectively (*p* = 0.12). Although found to be insignificant against placebo, the trial however provided insight into patient enrolment as it was noted that children seemed to respond better than the adults (Kurtzberg *et al*. 2010) and earlier intervention rather than a delayed treatment yielded better result. Following this a further adapted trial was carried out in 2017 (NCT02336230). This trial focused on adolescents and took patients from the age range of two months to 17 years at a grand total of 60 in the cohort. This trial was successful by improving 28-day response in paediatric patients by 70% with severe GVHD compared to the 45% in the 2009 trial. These data, alongside the 180-day safety, are expected to be granted an accelerated FDA approval, as of yet there are no publicly available data showing Prochymal distribution throughout Canada or New Zealand where it is licensed for the treatment of paediatric steroid-refractory GVHD. Further work in Japan has developed the therapy with a price point of £170,000, this is likely to have some impact as to why these therapies haven’t been seen to role out across the US and other countries. Drug affordability is a huge measure when determining drug license especially on non-profit healthcare systems seen in the US and Europe. In efforts to make these expensive therapies available for more patients, a new scheme was rolled out which enabled any drug eligible for regenerative medicine advanced therapy (RMAT) to be fast tracked for FDA approval.

Although not every trial carried out yield’s success similar to Prochymal, they do each provide insight as to how better outcomes can be achieved, for example, the 2015 phase III trial using ASC’s (adipose stromal cells) in enterocutaneous fistular disease for Chron’s (NCT01541579). In this trial, patients were given up to 120 million cells by multiple injections around the fistular. The resultant data were significant 50% of patients achieving remission compared to the placebo group an annual check-up confirmed that these results were also sustained (Panés *et al*. 2018). This trial was distinct from many previous unsuccessful trials because of the route of delivery; the cells were delivered directly into the fistular by local injections, whereas previously cells were being delivered intravenously. This allowed for an influx of cells that would otherwise have been unachievable and proved the safety of delivery in such a way that could now be utilized in other models. Several *in vivo* trials have shown that the use of MSCs in rodent ischemic stroke models, the rodent showed improvement. Numerous clinical trials involving MSCs are investigating the therapeutic effects of neurodegenerative and cardiovascular conditions (Hsuan *et al*. 2016).

The number of newly designed MSC clinical trials expanded from 2006–2012 but has in more recent years since 2018 began to plateau. Such a trend is difficult to explain but may be due to limited success in achieving desired outcomes in earlier trials or costings. Table 1 summarizes the recent registered clinical trials using stem cells for the treatment of neurological conditions.

**Table 1:** Outline of clinical trials investigating the use of MSC in different neurological conditions

NCT number	Title	Status	Study results	Conditions	Characteristics	Completion date
NCT03632135	Standard chemotherapy vs. chemotherapy guided by cancer stem cell test in recurrent glioblastoma	Recruiting	N/A	Recurrent glioblastoma	Phase III	January 2022
NCT02824653	Allogenic bone marrow mesenchymal stem cells infusion in patients with steroid-refractory GVHD	Completed	N/A	GVHD	Phase I Phase II	December 2018
NCT02795052	Neurologic stem cell treatment study	Recruiting	N/A	Neurologic disorders, nervous system diseases, neurodegenerative diseases, neurological disorders, stroke, traumatic brain injury; cadasil, chronic traumatic; encephalopathy, cerebral infarction, cerebral ischemia, and 10 more	N/A	June 2021
NCT01982682	Allogeneic hematopoietic stem cell transplantation for high-risk hematologic malignancies using one haploidentical donor	Completed	Results available	Hematopoietic/lymphoid cancer	Phase II	March 2017
NCT00798811	High-dose chemotherapy with autologous stem cell rescue in paediatric high-risk brain tumours	Active	N/A	Brain tumour	Phase II	December 2021
NCT00179803	Stem cell transplant for high-risk central nervous system (CNS) tumours	Unknown	N/A	Glioblastoma, astrocytoma, pineoblastoma, rhabdoid tumor, and supratentorial neoplasms	Phase II Phase III	September 2009
NCT00002619	Chemotherapy followed by bone marrow or peripheral stem cell transplantation in treating patients with glioblastoma multiforme or brain stem tumours	Completed	Has results	Brain and central nervous system tumours	Phase II	April 2000
NCT01922908	Mesenchymal stromal cells for ischemic stroke	Withdrawn	N/A	Ischemic stroke	−	April 2018
NCT03384433	Allogenic mesenchymal stem cell derived exosome in patients with acute ischemic stroke	Completed	N/A	Ischemic stroke	Phase I	December 2019
NCT00875654	Intravenous stem cells after ischemic stroke	Completed	N/A	Ischemia stroke	Phase II	October 2017
NCT03291366	Mesenchymal stem cells in central nervous system injury 2017	Active, not recruiting	N/A	Central nervous system injury	Phase I Phase II	January 2022
NCT04063215	A clinical trial to determine the safety and efficacy of hope biosciences autologous mesenchymal stem cell therapy for the treatment of traumatic brain injury and hypoxic-ischemic encephalopathy	Recruiting	N/A	Traumatic brain injury	Phase I Phase II	August 2021

## THE POTENTIAL OF MICROFRAGMENTED ADIPOSE TISSUE IN REGENERATIVE DRUG DELIVERY THERAPEUTIC

The potential of adipose tissue has been explored through the use of various engineered biomaterials in research.

### Acellular approach

Acellular approaches to adipose tissue processes have been explored due to the advantage of removing harvesting and compatibility complications. An example of this is matrigel, which is an extract from Engelbreth-Holm-Swarm (EHS), this mouse tumour product has been used in initial studies but due to its nature is not suitable for clinical application (Gomillion and Burg 2006). Initial work combined matrigel with the basic fibroblast growth factor (bGFG) subcutaneously in mice and resulted in vascularized coherent tissue containing mature adipocytes (Kawaguchi *et al*. 1998). The addition of bGFG compared to matrigel alone supported the release of microspheres (Kimura *et al*. 2002). These studies demonstrate the ability to exploit these physiological mechanisms of tissue formation, but it is important to note that the expansion of tissue cannot be controlled in regard to shape and dimensions. In attempts to rectify this issue, Walton *et al*. used a silicon dome in a rat model, filling the dome with matrigel and bFGF led to adipose tissue formation contained within the structure. This approach shows that precise control can be achieved however the process involves complex surgery followed by explanation when the structures are non-degradable (Walton *et al*. 2004).

### Cell-based approach

Scaffold implantation involved cells being seeded on prefabricated porous scaffold which can then be delivered by implantation. This technique means that shape and definition can be achieved however the process is invasive and requires surgery rather than being injectable. By far the most common synthetic biodegradable polymers used in clinic are polylactic acid (PLA) and polyglycolic acid (PGA) (Göpferich 1996). The first successful study saw PGA scaffolds seeded with ASC producing vascularized adipose tissue after five weeks of implantation into rats (Patrick *et al*. 1999). However, this scaffold was not successful long term with the whole structure being reabsorbed by five months. It has further been discovered that the time of reabsorption is proportional to the scaffold degradation.

Collagen offers another example of scaffolding advantageous for its cost and availability but hampered due to its quick degradation rates compared to PGA structures. Early into collagen studies, it was discovered that tissue differentiation was dependent on pore size of the scaffold (von Heimburg *et al*. 2001). Fat tissue has been demonstrated in nude mice following subcutaneous implantation with collagen spongers over six and 24 weeks (Tsuji *et al*. 2009).

### Cell-encapsulated hydrogels

These injectable cell carriers are advantageous as they are minimally invasive as an injection thereby reducing costs. In these cases, the cells are encapsulated during formation allowing for tight control of dimension used to fill shaped sites.

**Figure 2:**
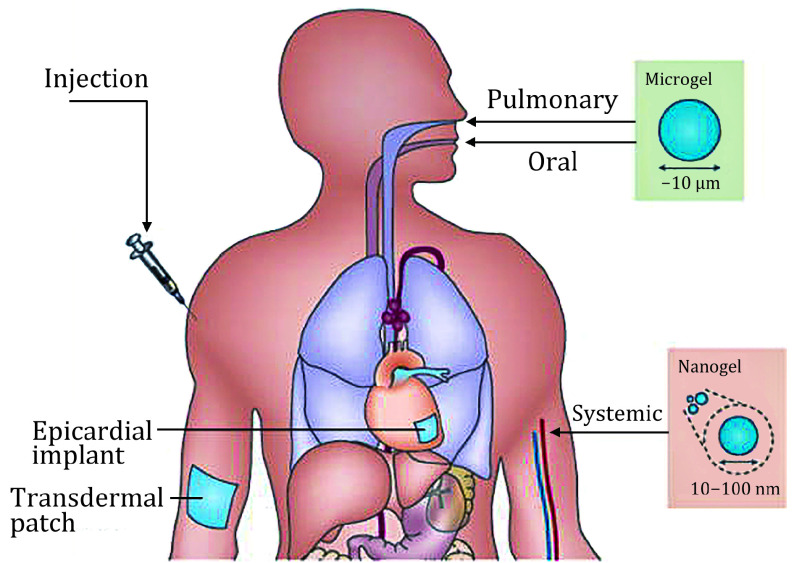
Diagram showing the different delivery routes in which hydrogels can be used (Li and Mooney 2016)

Figure 2 is the diagram of different delivery routes in which hydrogels can be used. An example of this type of tissue engineering is Fibrin, this natural polymer has been shown to form adipose tissues and has been used in cartilage redevelopment (Eyrich *et al*. 2006). Fibrin gel combined with ASCs have been injected into spaces beneath PLA structures in mice, leading to adipose tissue formation which was maintained after six weeks (Cho *et al*. 2005).

### Stem cells

There is great interest in using stem cells as carriers of therapeutic to target tissues and organs for treatment. Combined delivery of cells together with various information molecules has the potential to enhance, modulate or even initiate repair processes. The capability of stem cells to home and target inflammatory sites justifies their use as delivery agent for regenerative medicine purposes. One particular tissue of interest is adipose tissue. Adipose tissue has many unique properties, in the human body it is the most abundant tissue formulated of two specialized connective tissues; white adipose tissue (WAT) and brown adipose tissue (BAT). Currently the majority of research focusses on WAT tissue which is located throughout the body with major deposits in the abdomen, buttocks and thighs making the tissue highly accessible (Gesta *et al*. 2008). Additionally, on a cellular level WAT consists of a high volume of mature adipocytes as well as further important components including mesenchymal stem cells, pericytes and smooth muscle cells. These mesenchymal stem cells have the capacity of further proliferation and expansion when exposed to stimulation. Furthermore, adipose tissue has an extremely high metabolic rate and continuously undergoes remodelling supported by its surrounding capillary network. This expansion is supported by hypertrophy and hyperplasia (Christiaens and Lijnen 2010).

Support over recent years has been established for using adipose mesenchymal stems which have been isolated from fat tissue through liposuction. The advantages to these particular stem cells and procedure is that there is very little or non-apparent side effects (Nava *et al*. 2019). Currently there are two ways to retrieve adipose tissue and this is through enzymatic digestion or through mechanical force. The Enzymatic digestion of adipose tissue until recently was the go-to procedure until new technologies and knowledge allowed for other methods to come about. Enzymatic digestion uses different enzyme which include collagenase, trypsin and dispases to breakdown the adipose tissue to form stromal vascular fraction which is either cryopreserved or expand in culture. This method can be expensive and has potential impact of safety and efficiency (Oberbauer *et al*. 2015; Tremolada *et al*. 2016).

More recently non-enzymatic isolation has been developed through a mechanical force which separate the cells and aggregate formation. This method is expensive but allows a sterile field through a closed field.

There are several devices of interest to make MFAT (Table 2). Single used kits include Fatstem, Mystem, Lipocube and Lipogems (Trivisonno *et al*. 2019). First being Lipocell which uses a semipermeable memebrance to remove waste from the adipose tissue. This device acts in the same way as a diaysis casstte. It allows for a great regerneratve potential. This sytem is a closed system, sterile, fast and simple with mimimal transformation needed (Roato *et al*. 2020).

**Table 2:** The benefits and negatives of the different ways to extract adipose tissue from bench to bedside

Device	Method	Positive	Negative
Mechanical isolation (Lipogems and Lipocell)	Minimal invasive procedure (liposuction) usually fat tissue taken from the abdomen	Minimally invasive FDA approved Quick treatment Reduce need for major intervention Enzyme-free	Expensive

Enzymatic digestion	Lipo-aspiration	Efficient Yields higher cell counts	Expensive Prolongs the isolation time External compounds add (enzymes) Enzymatic method may be too aggressive and might destroy exosomes during processing Enzymatic treatment of cells digests the extracellular matrix surrounding the cells, possibly affecting cell secretory functions Might damage the cells, affecting cell function and viability

**Figure 3:**
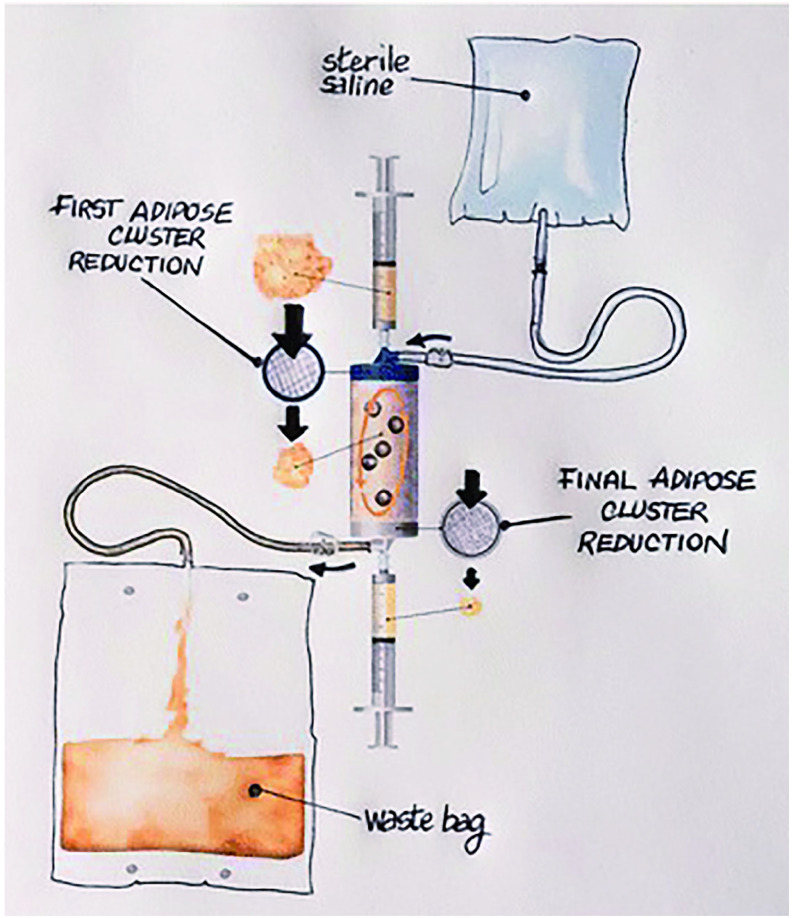
Schematic of the Lipogems device that is used to transform the adipose tissue into micro-fragmented adipose tissues (Tremolada *et al*. 2016)

Another particular enclosed device of interest is Lipogems. Lipogems is an innovative regenerative cellular technology that utilizes the patient’s own fat (adipose) tissue and administers via injection into an area for repair of injured or damaged tissue (Randelli *et al*. 2016). The process uses MFATs that allows for preservation of the structural niche. This preservation allows for transformations of pericytes into MSCs that initiates the regenerative process and releases anti-inflammatory factors. This is why Lipogems is often described as a "time-release" medium (Acosta *et al*. 2015). Figure 3 is the schematic of the Lipogems device that is used to transform the adipose tissue into micro-fragmented adipose tissues (MFATs). This technology shows promise in developing successful treatments in translational medicine due to the ease of collecting large quantities of adipose tissue with guaranteed substantial amount of MSCs which can be utilized to deliver specific drugs directly to sites with little to no side effects to surrounding tissue (Zeira *et al*. 2018). The technology has already shown great promise in cardiovascular disease, with studies showing the ability of this technology to repair the myocardium after exposure to synthetic agents (Koton *et al*. 2013). Systemic delivery of Lipogems has been shown to be beneficial for treatment of arthritis in joints of dogs. The work of Dr Giulio Alessandri has already demonstrated the success in using the technology to inhibit tumour growth *in vitro* (Coccè *et al*., 2017). Further to this, clinics have been offering lipogems as an injectable tissue therapy for the treatment of many different problems relating to the joints, to name some are tendon, ligaments, joints and muscle. Research has been conducted into this treatment with one paper looking at the safety and efficacy of using autologous, micro-fractured adipose tissue which has been extracted using a minimal invasive method for the treatment refractory knee osteoarthritis (OA). In fact, by the end of this research improvements in pain and the joint movement was noticed for 12 months and it has shown that this could be a non-surgical option in the future with more research needed (Panchal *et al*. 2018).

## POTENTIAL IMPACT OF AGING ON THE EFFICIENCY OF CELL THERAPIES FOR STROKE

The principal risk factor for stroke is undoubtedly age, with over half of all strokes occurring in men and women over 75 and a further 1/3 occurring in those over 85 (Boehme 2017). This leaves the entire older population at risk of stroke and whilst there are associated age changes that show variability due to genetics and lifestyle factors there are much more important sex differences in stroke with far more male cases per year (Popa-Wagner 2020).

These effects have been explored in previous models; one extensive study explored the recovery of neurological function following such ischemic event. Buchold *et al*. found that recovery in older rats following stroke was hardly detectable compared with the recovery of younger rats. In attempts to detect the reasoning for the slower recovery, Buchold *et al*. allowed the rats to recover in enriched areas and found significant improvements correlating with fewer proliferation astrocytes and smaller glial scars.

**Figure 4:**
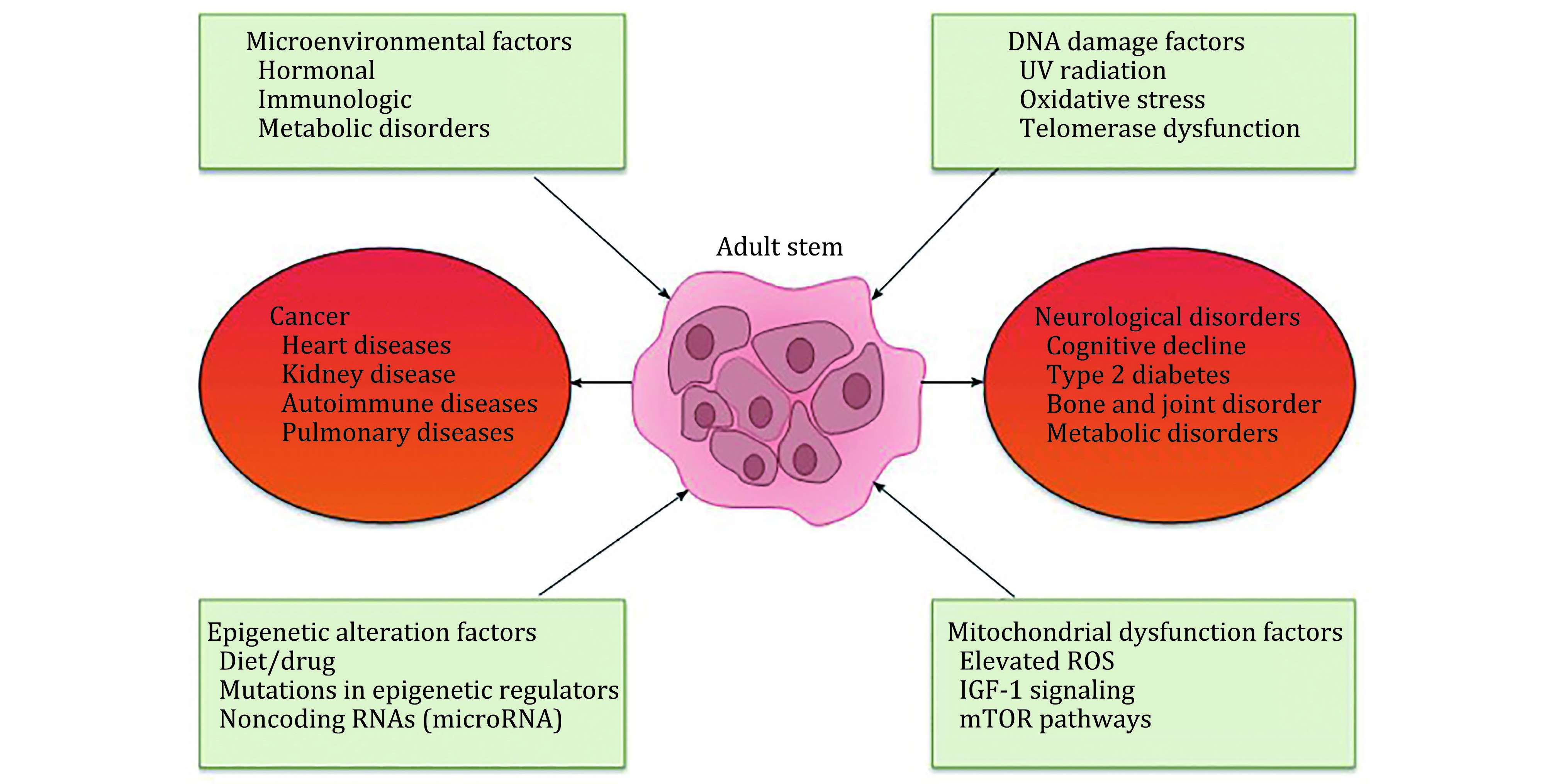
Potential mechanisms which drive differentiation of stem cells and diseases associated with age (Ahmed *et al*. 2017)

Age continues to be a limiting issue in therapy also, as we age so to do our cells and there are notably a smaller number of neural stem/progenitor cells and bone marrow MSCs in the elderly, this causes a decrease in stem cell functionality that leads to a decline in tissue rejuvenation, repair and cellular senescence (Koyuncu *et al*. 2015; Ahmed *et al*. 2017) (Fig. 4). These changes are also thought to affect biological features of the cells too resulting in decreased proliferation and increased senescence and apoptosis. Experimental models of stroke have developed effective neuroprotective strategies that once clinically translated have failed. The discrepancies may lie in the experimental studies as they are carried out on young adults which will not fully represent the true effect on an aged brain. An aged brain is typically categorised by increased astrocytic and microglial reactivity and decreased activity in plasticity related genes (MAP1B and βAPP). These such characteristics hinder response to therapeutic interventions for stroke. Potential to overcome this is the use of patient specific production of induced pluripotent stem cells. Tatarishvili *et al*. concluded that the use of neurons which had been generated from human skin-derived iPSC as a potential positive effect in the improved recovery of stroke specialised in the older age (Tatarishvili *et al*. 2014).

From a clinical standpoint, the use of iPSCs to facilitate the survival of neuronal environment is promising since they lack ethical concerns and graft rejection (Popa-Wagner *et al*. 2014). However, it is still unknown how the aged brain will respond, so much more work is needed to fully understand the mechanisms.

## SUMMARY AND PERSPECTIVES

This mini review focuses on looking at the potential use of adipose-derived stem cell preparations in neurological targeted drug delivery, repair, and regeneration. Over many years the development and knowledge for the use of stem cells in regenerative medicine and repair tissue have been greatly researched. MSC has a great potential due to the ease at which this stem cells can now be harvested through a minimal invasive procedure with the adipose tissue being fragmented with a special device known as Lipogems and well as other single use devices such as Fatstem, Mystem, and Lipocube.

## Conflict of interest

Laura Combes, Xenia Sawkulycz, Wen-Hui Fang, Baoqiang Guo and Mark Slevin declare that they have no conflict of interest.
